# Supplementary material for: An Ultra-Sensitive and Multifunctional Electronic Skin with Synergetic Network of Graphene and CNT
Source: Nanomaterials (Basel). 2022 Dec 30;13(1):179. doi: 10.3390/nano13010179 (PMC9823652; doi:10.3390/nano13010179)
Supplement: Supplementary file 1 [file nanomaterials-13-00179-s001.zip › nanomaterials-2096929-supplementary.pdf]

nanomaterials

Supporting Information

An Ultra-Sensitive and Multifunctional Electronic Skin with Synergetic Network of Graphene and CNT

Yu Wang [1,2,†], Tian-Rui Cui [1,2,†], Guang-Yang Gou [1,2], Xiao-Shi Li [1,2], Yan-Cong Qiao [1,2], Ding Li [1,2], Jian-Dong Xu [1,2], Yi-Zhe Guo [1,2], He Tian [1,2,*], Yi Yang [1,2,*] and Tian-Ling Ren [1,2,3,*]

[1] School of Integrated Circuit, Tsinghua University, Beijing 100084, China
[2] Beijing National Research Center for Information Science and Technology (BNRist), Tsinghua University, Beijing 100084, China
[3] Center for Flexible Electronics Technology, Tsinghua University, Beijing 100084, China
* Correspondence: tianhe88@tsinghua.edu.cn (H.T.); yiyang@tsinghua.edu.cn (Y.Y.); rentl@tsinghua.edu.cn (T.-L.R.)
† These authors contributed equally to this work.

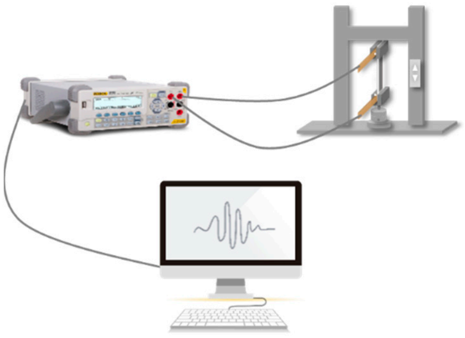

Figure S1. The test system diagram.

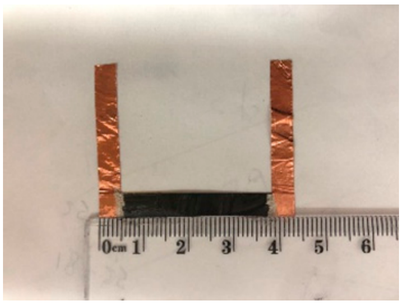

Figure S2. The digital picture of the SCG e-skin.

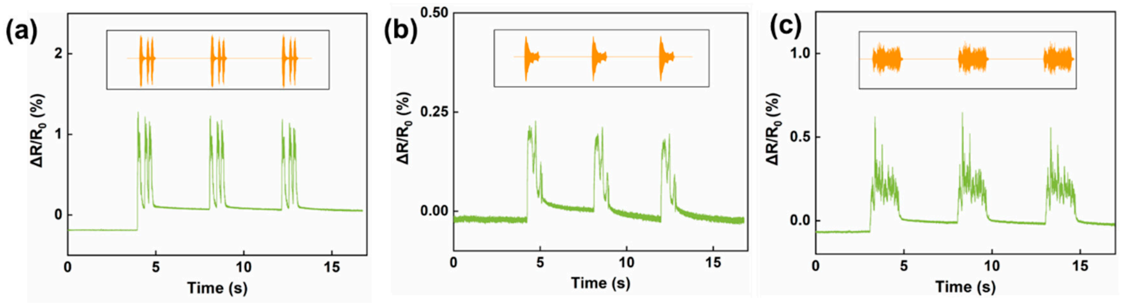

Figure S3. The comparison between the initial audio wave of animal sound and the corresponding sound vibration signal. (**a**) meow (**b**) bark (**c**) cock crow.

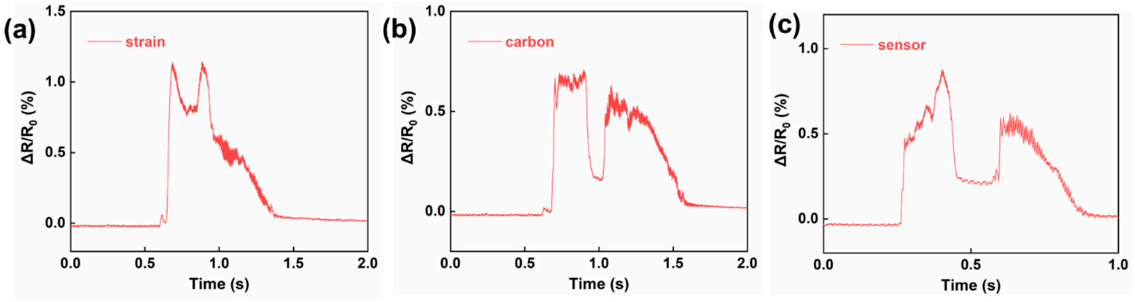

Figure S4. The response towards English words from a loudspeaker. (**a**) ″strain″ (**b**) ″carbon″ (**c**) ″sensor″.

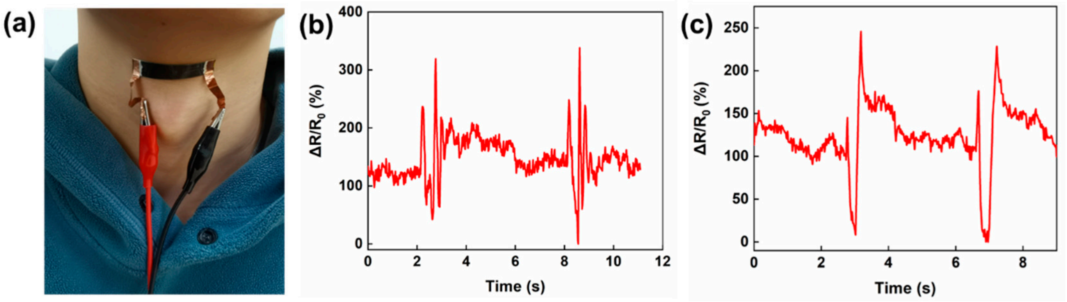

Figure S5. Throat muscle movement detected by SCG e-skin attached to the throat. (**a**) Tester with a SCG e-skin attached to the throat. (**b**) nod (**c**) shake head.
